# Reliability and validity of the German version of the DePaul Symptom Questionnaire Post-Exertional Malaise (DSQ-PEM)

**DOI:** 10.3389/fpsyt.2025.1647040

**Published:** 2025-09-04

**Authors:** Charlotte Kuczyk, Mariel Nöhre, Christoph Herrmann-Lingen, Maike Stolz, Christian Krauth, Elmar Brähler, Leonard A. Jason, Martina de Zwaan

**Affiliations:** ^1^ Department of Psychosomatic Medicine and Psychotherapy, Hannover Medical School, Hannover, Germany; ^2^ Department of Psychosomatic Medicine and Psychotherapy, University Medical Center Göttingen, Göttingen, Germany; ^3^ Institute for Epidemiology, Social Medicine and Health Systems Research, Hannover Medical School, Hannover, Germany; ^4^ Center for Health Economics Research (CHERH), Leibniz University Hannover, Hannover, Germany; ^5^ Department of Psychosomatic Medicine and Psychotherapy, University Medical Center of the Johannes Gutenberg-University, Mainz, Germany; ^6^ Department of Medical Psychology and Medical Sociology, University Medical Center of Leipzig, Leipzig, Germany; ^7^ Center for Community Research, DePaul University, Chicago, IL, United States

**Keywords:** DSQ-PEM, validation, psychometric properties, post-exertional malaise (PEM), reliability and validity

## Abstract

**Objective:**

This study aimed to examine the psychometric properties (reliability and validity) of a German version of the DSQ-PEM, using a representative sample from the German general population (final n = 2,263) and a clinical sample with diagnosed post-COVID-19 condition (PCC) (final n = 1,448).

**Methods:**

The internal consistency of the German version of the DSQ-PEM was calculated separately for both samples using Cronbach’s alpha. Convergent validity was assessed in both samples through correlations between the DSQ-PEM and the Patient Health Questionnaire (PHQ - 4), and for the PCC sample, additionally through a correlation between the DSQ-PEM and the Chalder Fatigue Scale. To evaluate known-group validity, differences in DSQ-PEM scores between the general population sample and the PCC sample were analyzed, adjusting for relevant sociodemographic variables. Additionally, gender- and age-related differences in the DSQ-PEM were calculated separately for both samples.

**Results:**

The DSQ-PEM items demonstrated excellent internal consistency in both the general population and PCC samples. Higher DSQ-PEM scores correlated with increased symptoms of anxiety and depression in both samples and were also associated with higher scores on the Chalder Fatigue Scale in the PCC sample, indicating good convergent validity. The known-group validity analyses revealed that the German version of the DSQ-PEM effectively differentiates between individuals from the general population and those with PCC, even after adjusting for relevant sociodemographic variables. Advanced age and female gender were associated with higher DSQ-PEM scores in the general population sample. No such correlation was found in the PCC sample.

**Conclusion:**

In summary, this study confirms the strong psychometric properties of the German version of the DSQ-PEM and supports the instrument as a reliable and valid tool for measuring PEM in Germany and other German-speaking countries.

## Introduction

Chronic Fatigue Syndrome (CFS), also known as myalgic encephalomyelitis (ME), is a persistent medical condition characterized by a complex array of severe somatic symptoms. These symptoms include profound fatigue, muscular weakness, pain, cognitive impairments, sleep disturbances, flu-like manifestations, and orthostatic intolerance.

The pathophysiological mechanism of CFS remains unknown; however, numerous studies suggest dysregulation of the immune system (1–3). While genetic factors may play a potential role in the development and progression of CFS (4), viral infections are considered the most common triggers of the disease. Consequently, CFS has received renewed scientific and clinical attention, particularly in the context of the COVID - 19 pandemic. A meta-analysis conducted during the pandemic estimated the global prevalence of CFS to be around 0.89%, with prevalence figures varying depending on the case definition used (5).

The definition “post-COVID-19 condition” (PCC) encompasses a range of symptoms and signs experienced by patients following COVID - 19 infection, alongside an initial infection by SARS-CoV-2 (6). Some studies suggest that the prevalence of CFS has risen since the onset of the COVID - 19 pandemic. Other studies also postulate that severe fatigue is the most common symptom in patients with long COVID and that it has pathological similarities to CFS (7). Furthermore, many patients with PCC meet the diagnostic criteria for CFS (8, 9); however, this does not apply to all patients with PCC.

A primary symptom of CFS is post-exertional malaise (PEM), as shown in a population-based study (10). This research indicated that PEM is the most significant distinguishing characteristic between healthy controls and individuals who meet the criteria for CFS (11). PEM manifests as an inappropriate loss of physical and mental resilience, characterized by significant muscular and cognitive fatigability after only minor physical, cognitive, emotional, or orthostatic stress. PEM can occur up to 72 hours after exercise and can last for 24 hours or longer. A recent investigation into the symptoms of CFS in patients experiencing Long COVID revealed that PEM was the most frequently reported symptom in patients with PCC, demonstrating a prevalence of 21.9% (9).

Currently, there are no recognized biomarkers or established diagnostic tests to assess CFS. Consequently, CFS is diagnosed clinically in accordance with established diagnostic criteria, with the Canadian Consensus Criteria (CCC) (12) being commonly utilized for this purpose.

In this context, the DePaul Symptom Questionnaire for Post-Exertional Malaise (DSQ-PEM) was developed with active participation and guidance of patients as a valid and reliable psychometric instrument for assessing PEM (13). The first five PEM-related items of the DSQ-PEM have accurately identified 97% of patients with CFS. The original DSQ-PEM items exhibit strong overall psychometric properties, including high test-retest reliability, construct validity, predictive validity, sensitivity and specificity, and discriminant validity (10, 14). The DSQ-PEM has already been translated into numerous languages and is utilized in many countries.

To the best of our knowledge, however, no authorized and rigorously validated German version of the DSQ-PEM is currently available. Therefore, the aim of this study was to validate a German version of the DSQ-PEM in a large, representative German population sample using an elaborate pre-translation and back-translation procedure. This process is designed to develop an appropriate tool for screening in clinical practice and for evaluating the symptom burden over time in patients with (CFS) in Germany and other German-speaking countries.

Given that higher levels of PEM symptoms have been associated with higher levels of depression in previous studies (15, 16), a questionnaire to assess anxiety and depression was employed to test the convergent validity. To examine known-group validity, the study assessed how effectively the German version of the DSQ-PEM differentiates between a large, representative German population sample and a physician-diagnosed PCC sample reporting persistent symptoms.

## Materials and methods

### General population sample

The PEM data were collected as part of a representative cross-sectional survey on physical and mental well-being in the German resident population. In this context, a random sample of the entire German population (minimum age 16 years) was obtained from March to July 2024. Since there is no directory containing the addresses of all private households or individuals in Germany, the “ADM Sampling System for Face-to-Face Surveys” was employed to collect a representative sample of the German population. A market and social research company (USUMA GmbH, Berlin) conducted the acquisition of respondents and face-to-face interviews with trained interviewers. Initially, a sample of N = 6,895 individuals was contacted. Due to quality-neutral dropouts (e.g., unoccupied homes; no eligible person from the population in the household) or systematic dropouts (e.g., households not visited despite repeated attempts; target person ill and unable to participate in the interview; target person refuses the interview), interviews were conducted based on a sample of N = 2,559 individuals. After excluding unusable interviews, data from N = 2,504 individuals were available. Of these, those who had not yet reached the age of 18 (N = 50, 2.0%) and those who stated in the survey that they had been diagnosed with PCC (N = 168, 6.7%) were subsequently excluded, leaving a final sample of N = 2,263 for the statistical analyses.

The population-based survey adhered to the ethical guidelines of the International Code of Marketing and Social Research Practices, established by the International Chamber of Commerce and the European Association for Opinion and Market Research. All participants participated voluntarily and provided their written informed consent before their inclusion (ethical approval 008/24-ck from the University of Leipzig).

### PCC validation sample

Individuals diagnosed with PCC were identified using health insurance data from the largest statutory health insurer in Lower Saxony (AOK Niedersachsen), which provides coverage for approximately one-third of the adult population in this region, as reported in the VePoKaP study (https://doi.org/10.1371/journal.pone.0316335) funded by the Ministry of Science and Culture of Lower Saxony in Germany (14 - 76403-184). Subsequently, individuals insured with AOK Niedersachsen who satisfied the following inclusion criteria were invited via mail to participate in an online survey: 1) Individuals aged 18 years or older with a confirmed PCC diagnosis, indicated by the ICD code U09.9! in their claims data (outpatient billing records or a certificate of incapacity for work from 2022), 2) residents of Lower Saxony, and 3) individuals insured with AOK Niedersachsen without interruption since 2019. Patients under legal care and AOK employees were excluded. A total of N = 26,438 individuals were deemed eligible, from whom, for budgetary reasons, N = 20,163 were selected and contacted by mail. Of these, N = 2,159 (10.7%) individuals provided their consent and completed the survey.

Given that previous research has indicated that certain patients with PCC meet the clinical criteria for CFS (17), individuals whose diagnosis was made less than one year ago or who reported no longer having PCC symptoms were excluded from the formation of a corresponding selective sample characterized by persistent, fatigue-dominant PCC prior to statistical analysis (N = 486; 22.5%). Since the Chalder Fatigue Scale is a short and reliable instrument for assessing fatigue in the general population (18), patients who scored ≤ 4 points on the Chalder Fatigue Scale (CFQ - 11) were also excluded (N = 276; 12.8%) (7, 19). Thus, N = 1,448 individuals with fatigue-dominant PCC were included in the subsequent statistical analyses.

Ethical approval for the collection of the PCC validation sample was obtained from the ethics committees of Hannover Medical School (reference number 11077_BO_K_2023). Participants provided their informed consent electronically.

### Measures

#### DSQ-PEM

The entire DSQ underwent factor analysis, during which the PEM items were discerned as a distinct factor (13). The original DSQ-PEM (13) was translated into German (questionnaire available in the **Supplementary File**). To ensure accuracy and identify potential ambiguities or misunderstandings in the initial translation, a back-translation was conducted by an independent, licensed translation agency. This back-translation was reviewed by one of the American authors of the original scale (L.A.J.) for any discrepancies with the original version. The DSQ-PEM includes five items specifically aimed at measuring PEM: “Dead, heavy feeling after starting to exercise,” “Next day soreness or fatigue after non-strenuous, everyday activities,” “Mentally tired after the slightest effort,” “Minimum exercise makes you physically tired,” and “Physically drained or sick after mild activity.” The frequency and severity of these five items are assessed in relation to the last six months. Frequency is rated on a 5-point Likert scale: 0 = none of the time, 1 = a little of the time, 2 = about half the time, 3 = most of the time, and 4 = all of the time; the severity of each symptom is rated on a 5-point Likert scale of 0 = symptom not present, 1 = mild, 2 = moderate, 3 = severe, and 4 = very severe. In addition, the DSQ-PEM contains three additional items that record the duration of symptom worsening after activity. The first two items feature the questions: “Do you experience a worsening of your fatigue/energy-related illness after engaging in minimal physical effort?” and “Do you experience a worsening of your fatigue/energy-related illness after engaging in mental effort?” This is followed by a question on the duration of PEM: “If you feel worse after activities, how long does this last?” The participants’ answers regarding the duration of PEM are coded as follows: 1 = < 1 h, 2 = 2 – 3 h, 3 = 4 – 10 h, 4 = 11 – 13 h, 5 = 14 – 23 h, 6 = > 24 h. The fourth supplementary PEM item assesses how quickly patients recover from activities typically undertaken by healthy individuals. The question is: “If you were to become exhausted after actively participating in extracurricular activities, sports, or outings with friends, would you recover within an hour or two after the activity ended?” The DSQ-PEM has demonstrated good reliability in terms of test-retest reliability, and the first five DSQ-PEM items also exhibit good internal consistency (α = 0.84) (20). The various evaluation options available for the DSQ-PEM are delineated below:

Binary PEM Scores: Regarding the evaluation of the DSQ-PEM, several evaluation options have been described so far, and all these options were considered in the present validation study. One possible evaluation option consists of two “scoring steps.” Scoring step 1 checks whether at least one of the first five DSQ-PEM items exceeds a predefined threshold. An item exceeds the threshold value if it achieves a scale value between 2 – 4 in terms of severity (corresponding to “moderate,” “severe,” or “very severe”) and at the same time a scale value of 2 – 4 in terms of frequency (corresponding to “about half the time,” “most of the time,” or “all of the time”). Additional items regarding recovery time, symptom exacerbation due to physical activity, and the duration of PEM are scored as part of “Scoring Step 2.” The following applies: at least one positive answer (“yes”) to item 7 or 8, “do you experience a worsening of your fatigue/energy-related illness after engaging in minimal physical and/or mental effort?” is required, and a PEM duration of ≥14 hours must be specified to be considered criterion-fulfilling. Using the supplementary five DSQ PEM items, and in particular the duration of symptoms in the “Scoring Step 2”, Cotler et al. (2018) (13), could demonstrate that people with ME and CFS can be differentiated from those with at least two other fatiguing illnesses, multiple sclerosis and post-polio syndrome.

Continuous PEM scores: A sum of frequency and severity is calculated for each of the first five items. Based on the 5-point Likert scale, a score ranging from 0 to 8 is determined for each of the five items.

Extended PEM total score: To calculate an extended PEM total score, the frequency and severity of the first five items (maximum score of 40), along with the duration of the PEM, are taken into account. The duration of the PEM receives a score between 0 and 6, allowing for a maximum possible score of 46.

#### PHQ-4

The Patient Health Questionnaire for Depression and Anxiety (PHQ - 4) (21–23) was used to assess convergent validity. The PHQ - 4 was chosen for its conciseness, robust psychometric characteristics, and widespread international use. Extensive research has verified its reliability, validity, and high concordance with clinical diagnostic interviews across diverse cultural settings, emphasizing its utility as a practical and well-established screening tool.

The frequency of occurrence of the following symptoms is evaluated: “Little interest or pleasure in doing things,” “Feeling down, depressed, or hopeless,” “Feeling nervous, anxious, or on edge,” “Not being able to stop or control worrying,” using a scale from 0 = “not at all” to 3 = “nearly every day.” The total score ranges from 0 to 12 accordingly. Previous studies have demonstrated strong internal consistency, with Cronbach’s alpha exceeding 0.80 for the PHQ - 4. In the present study, Cronbach’s α was 0.87 for both the general population sample and the PCC sample, indicating good internal consistency.

### Chalder-Fatigue-Scale

In the PCC sample, the Chalder Fatigue Scale (CFQ - 11) (18, 24, 25) was used to further characterize the PCC sample. The scale consists of 11 items rated on an ordinal scale from 0 to 3. In the present study, a bimodal evaluation procedure was employed in which the response options were dichotomized: responses with values 0 or 1 were coded as 0, while responses with values 2 or 3 were coded as 1. The resulting score ranges from 0 to 11. According to the conventional case definition outlined by Chalder et al. (1993) (24), a cut-off value of ≥ 4 on the bimodal scale is considered an indication of clinically relevant fatigue.

### Statistical analyses

Statistical analyses were conducted using IBM SPSS Statistics. Chi-square tests were employed for categorical variables and Mann-Whitney U tests for continuous variables in all analyses delineated below. A p-value <.05 was considered statistically significant. The effect sizes were interpreted according to the guidelines by Cohen (1988) (26). Descriptive statistics, including age, gender, and educational status (<12 years of schooling vs. ≥12 years of schooling), were calculated separately for both the general population sample and the PCC sample.

The following analyses were performed to assess the psychometric properties of the scale: To determine internal consistency, Cronbach’s α was calculated independently for each sample for the first five DSQ-PEM items, utilizing the continuous PEM scores. Convergent validity was examined in both samples using Pearson correlations between the extended PEM total score and the PHQ - 4 sum score. In the PCC sample, a correlation was also conducted with the total score of the Chalder Fatigue Scale.

To test known-group validity, differences between the general population sample and the PCC sample were analyzed für the binary DSQ-PEM scores (PEM screening positive vs. negative), the continuous PEM scores and the extended PEM total score.

For a more fine-grained analysis, additional regression analyses were carried out, including the continuous evaluation variables (continuous PEM scores, extended PEM total score) as dependent variables, along with the sample affiliation and relevant sociodemographic variables that emerged as significant in the context of the sample comparisons as independent variables.

Gender differences in binary PEM scores, continuous PEM scores and the extended PEM total score were conducted for both the general population sample and the PCC sample. Age-related differences in binary PEM scores across seven age groups (≤ 24, 25 - 34, 35 - 44, 45 - 54, 55 - 64, 65 - 74, and ≥ 75 years) in both samples. Differences among age groups concerning the continuous PEM scores and the extended PEM total score were evaluated using Kruskal-Wallis tests; Dunn-Bonferroni *post-hoc* tests were subsequently conducted to further clarify the group differences.

## Results

### Description of the samples

The sociodemographic characteristics of the samples are shown in **Table 1**. In the sample from the general population, the proportion of women was 51.3% (N = 1162), while in the PCC sample, it was 71.7% (N = 1038). The median age in the general population sample was 51 years (interquartile range [IQR] = 29), whereas in the PCC sample, it was 53 years (interquartile range [IQR] = 17). The two samples differed significantly in terms of gender and years of education.

**Table 1:** Comparison of sociodemographic data between samples.

Variables		General population sample (N = 2263)	PCC sample (N = 1448)	Statistics Chi-Square test (χ², df, p value)/Mann-Whitney U test (Z, p value)
Percent females	n (%)	1162 (51.3)	1038 (71.7)	χ² = 151.61 df = 2 p <.001
Age, years	Mean (SD)	49.92 (17.5)	51.08 (12.28)	Z = -1.82 p = .069
	Median (IQR)	51.0 (29)	53.0 (17.0)	
Educational level	< 12 years of school, n (%)	1691 (75.1)	1085 (83.5)	χ² = 34.09 df = 1 p <.001
	≥ 12 years of school, n (%)	562 (24.9)	215 (16.5)	

### Internal consistency

The initial five items of the DSQ-PEM demonstrated excellent internal consistency in both samples for the continuous PEM scores. In the general population sample, Cronbach’s a was measured at .94. In the PCC sample, Cronbach’s a was determined to be .91.

### Convergent validity

Pearson correlations indicated significant associations between the extended PEM total score and the PHQ - 4 sum score in the general population (r = .599, p <.001) as well as in the PCC sample (r = .471, p <.001). Additionally, in the PCC sample, the extended PEM total score was significantly correlated with the Chalder Fatigue Scale score (r = .621, p <.001).

### Known-group validity

The chi-square tests revealed that, considering the binary evaluation method, “positive screening” for PEM was significantly more prevalent in the PCC sample, concerning all individual items and scoring steps 1 and 2, compared to the general population sample (**Table 2**). Also, when treating the DSQ-PEM items as continuous evaluation variables (i.e., continuous PEM scores), higher values were noted for all items in the PCC sample relative to the general population (**Table 3**). The extended PEM total score was also significantly higher in the PCC sample compared to the general population (**Table 4**).

**Table 2:** Comparisons of binary PEM scores between the general population sample and the PCC sample (Scoring Steps 1 and 2).

	General population sample (n = 2263)	PCC sample (n= 1448)	Chi-Square test (χ², df, p value)
1. A minimum of exercise makes you physically tired, n (%)	196 (8.7)	1091 (76.2)	χ² = 1755.61 df =1, p <.001, φ = 0.69
2. Physically drained or sick after mild activity, n (%)	186 (8.3)	868 (60.7)	χ² =1176.63 df =1, p <.001, φ = 0.57
3. Next-day soreness or fatigue after non-strenuous, everyday activities, n (%)	128 (5.7)	876 (61.2)	χ²= 1359.61 df =1, p <.001, φ = 0.61
4. Mentally tired after the slightest exertion, n (%)	151 (6.7)	915 (63.9)	χ² = 1395.59 df =1, p <.001, φ = 0.62
5. Dead, heavy feeling after starting to exercise, n (%)	142 (6.3)	918 (64.2)	χ² = 1429.00 df =1, p <.001, φ = 0.62
Scoring Step 1, n (%)	286 (12.8)	1254 (87.7)	χ² = 2014.28 df = 1, p <.001, φ = 0.74
7 & 8. Do you experience a worsening of your fatigue/energy-related illness after engaging in minimal physical and/or mental effort? n (%)	675 (29.9)	1227 (85.7)	χ² = 1090.66 df =1, p <.001, φ = 0.54
9. Duration 14 – 23 hours or > 24 hours, n (%)	23 (1.1)	329 (23.0)	χ² = 458.78 df =1. p <.001, φ = 0.36
Scoring Step 2, n (%)	18 (0.8)	298 (20.8)	χ² = 448.50 df =1, p <.001, φ = 0.35

The figures n (%) indicate the number of positive screenings.

**Table 3:** Comparison of the continuous PEM scores between the general population sample and the PCC sample (0 – 8 for each item).

		General population sample (N = 2263)	PCC sample (N = 1448)	Mann-Whitney U test (Z, p value, r)
1. A minimum of exercise makes you physically tired	M (SD)	1.08 (1.66)	5.21 (1.88)	Z = 45.22 p <.001 r = 0.76
	Median (IQR)	0.00 (2.00)	6.00 (2.00)	
2. Physically drained or sick after mild activity	M (SD)	1.05 (1.67)	4.48 (2.07)	Z= 41.21 p <.001 r = 0.69
	Median (IQR)	0.00 (2.00)	5.00 (3.00)	
3. Next-day soreness or fatigue after non-strenuous, everyday activities	M (SD)	0.74 (1.42)	4.39 (2.14)	Z= 43.82 p <.001 r = 0.73
	Median (IQR)	0.00 (1.00)	4.00 (3.00)	
4. Mentally tired after the slightest exertion	M (SD)	0.82 (1.53)	4.57 (2.11)	Z= 43.92 p <.001 r = 0.73
	Median (IQR)	0.00 (2.00)	5.00 (3.00)	
5. Dead, heavy feeling after starting to exercise	M (SD)	0.78 (1.51)	4.60 (2.16)	Z= 44.08 p <.001 r= 0.74
	Median (IQR)	0.00 (2.00)	5.00 (3.00)	

**Table 4:** Comparison of the general population sample and the PCC sample with regard to the extended PEM total score (0 - 46).

	General population sample (N = 2263)	PCC sample (N = 1448)	Mann-Whitney U test (Z, p value)
Mean (SD)	6.25 (7.63)	26.42 (9.63)	Z = 44.47 p <.001
Median (IQR)	2.00 (8.00)	26.00 (15.00)	

Even when controlling for sociodemographic variables in the regression analyses, sample membership proved to be a significant predictor of the continuous PEM scores and the extended PEM total score, as significantly higher values were found in the PCC sample compared to the general population sample (**Table 5** and **Supplementary Table 1**).

**Table 5:** Results of the regression analysis, including the extended PEM total score as the dependent variable, along with sample membership and sociodemographic variables as independent variables.

Predictor/Moderator	B	SE	β	t	p
Sample	19.85	.30	.74	65.09	<.001
Sex	1.42	.30	.05	4.70	<.001
Age in Years	.10	.01	.12	10.92	<.001
Education	-.73	.35	-.02	-2.05	.040

### Comparison by gender and age groups

**Figure 1:**
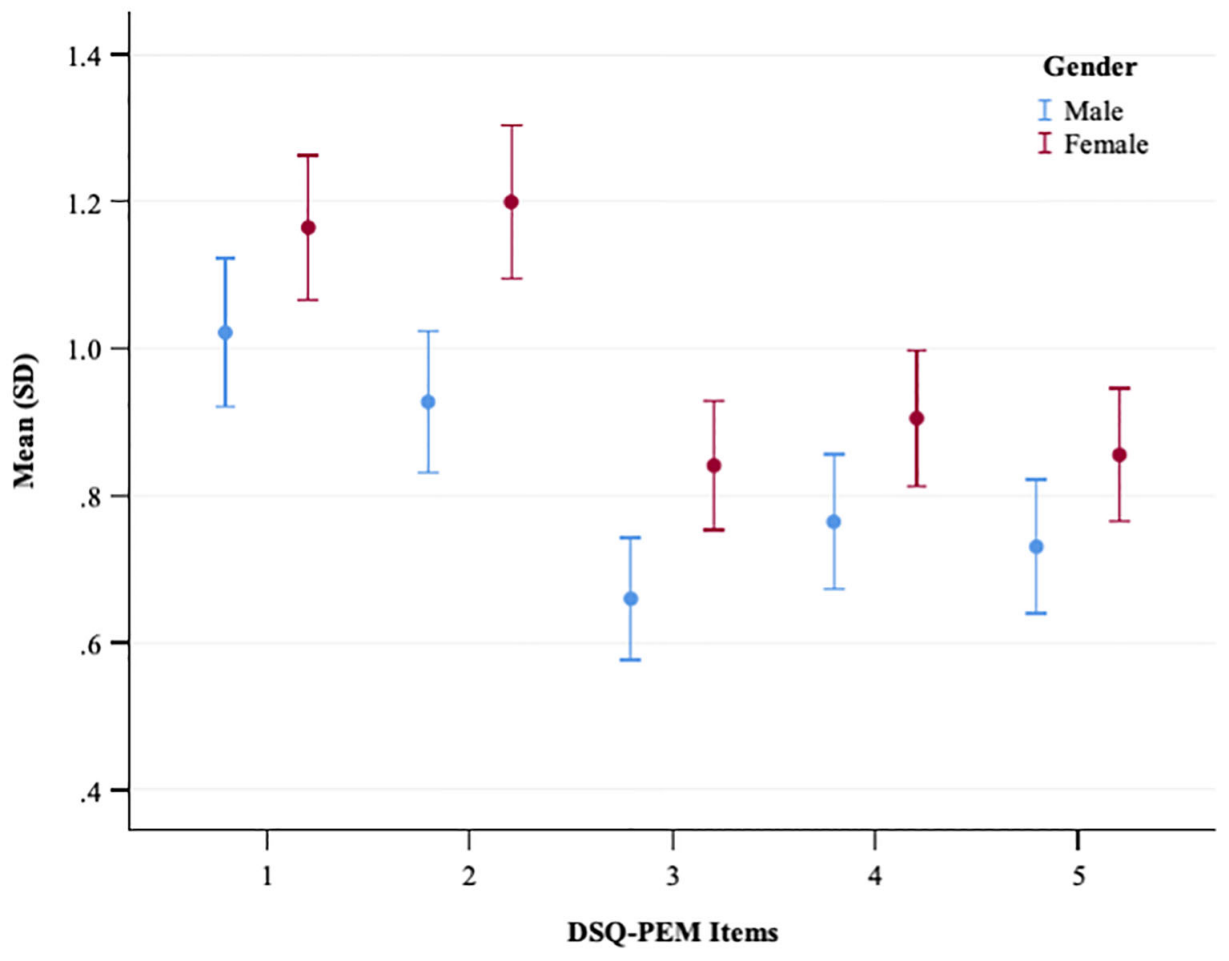
A plot showing the mean and standard deviation of DSQ-PEM items for males and females in the general population sample. The x-axis represents DSQ-PEM items numbered one to five, and the y-axis shows the mean score ranging from 0.40 to 1.40 (possible range 0-8). Male data points are in blue, female in red. Error bars display the variability in scores.

**Figure 2:**
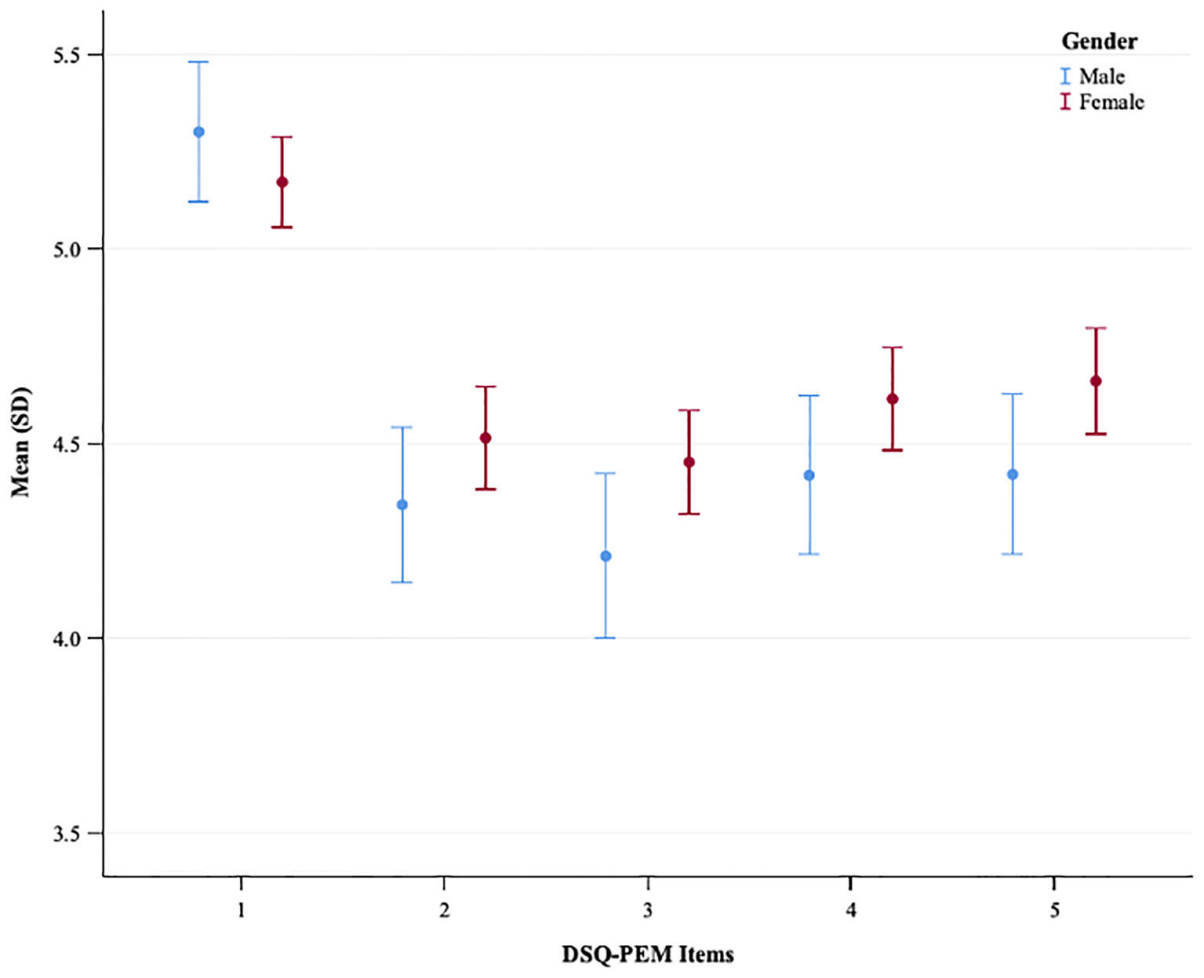
A plot showing the mean and standard deviation of DSQ-PEM items for males and females in the PCC sample. The x-axis represents DSQ-PEM items numbered one to five, and the y-axis shows the mean score ranging from 3.5 to 5.5 (possible range 0-8). Male data points are in blue, female in red. Error bars display the variability in scores.

The gender comparison indicated no statistically significant differences between males and females within the general population sample concerning the number of positive screenings in scoring steps 1 and 2 (**Supplementary Table 2**). Conversely, significant disparities were observed across all five continuous PEM scores and the extended PEM total score, with women showing notably higher values (**Figure 1**, **Supplementary Tables 3, 4**). In the PCC sample, gender-based differences were less prominent (**Figure 2**, **Supplementary Tables 5, 6**); however, women again exhibited significantly elevated values for the extended PEM total score (**Supplementary Table 7**).

**Figure 3:**
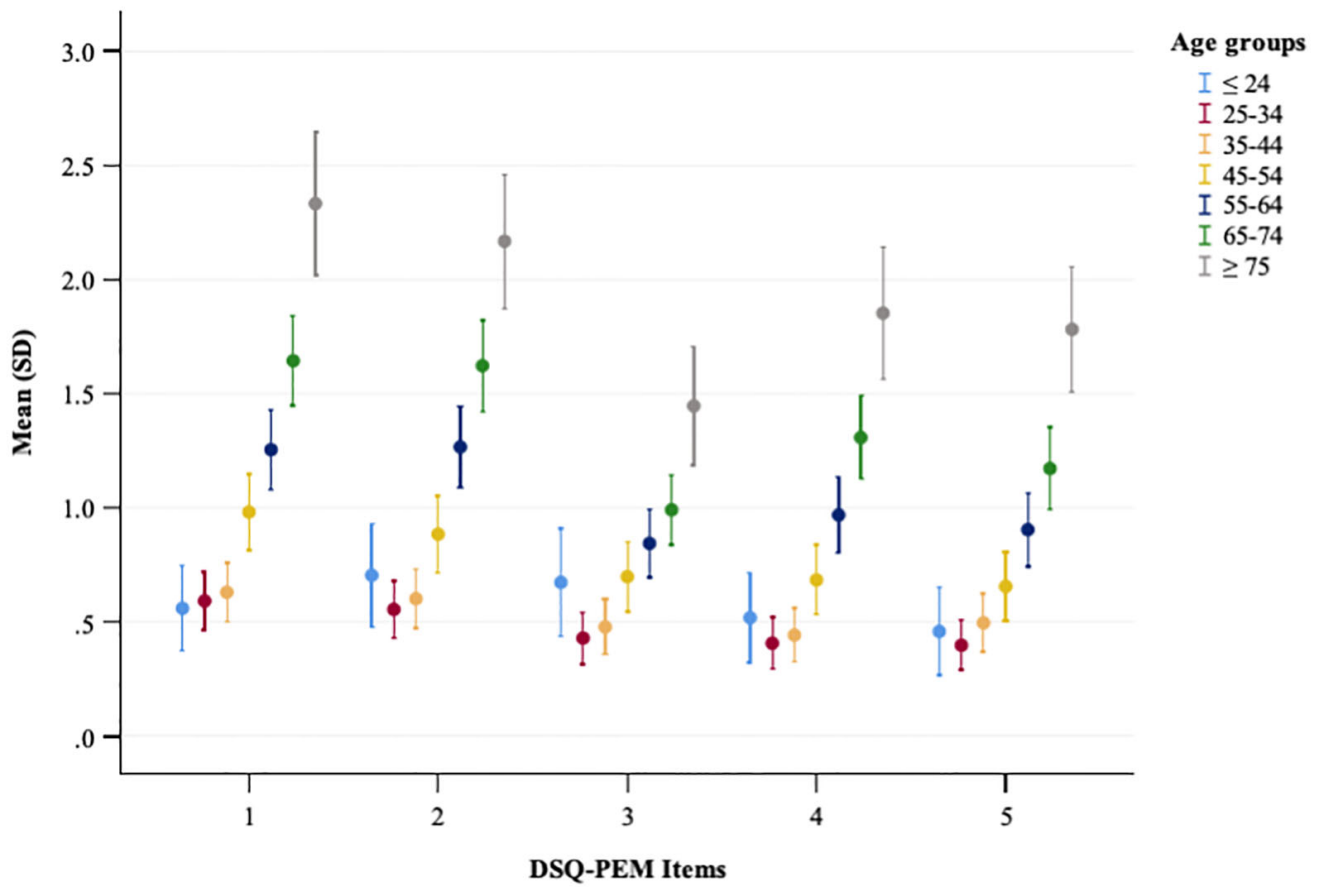
A plot showing the mean and standard deviation of DSQ-PEM items across different age groups in the general population sample. The x-axis represents DSQ-PEM items numbered one to five, and the y-axis shows the mean score ranging from 0 to 3.0 (possible range 0-8). Error bars display the variability in scores.

In the general population sample, chi-square analyses revealed significant differences among age groups with regard to the number of positive screenings in scoring steps 1 and 2 (**Supplementary Table 8**). Additionally, Kruskal-Wallis tests identified significant distinctions among age groups for all five continuous PEM scores and the extended PEM total score (**Supplementary Tables 9, 10**). Overall, PEM levels increased with age (**Figure 3**). *Post-hoc* analyses employing the Dunn-Bonferroni method confirmed that nearly all pairwise age groups differed significantly from one another (p <.001), with the exception of a few adjacent age groups.

**Figure 4:**
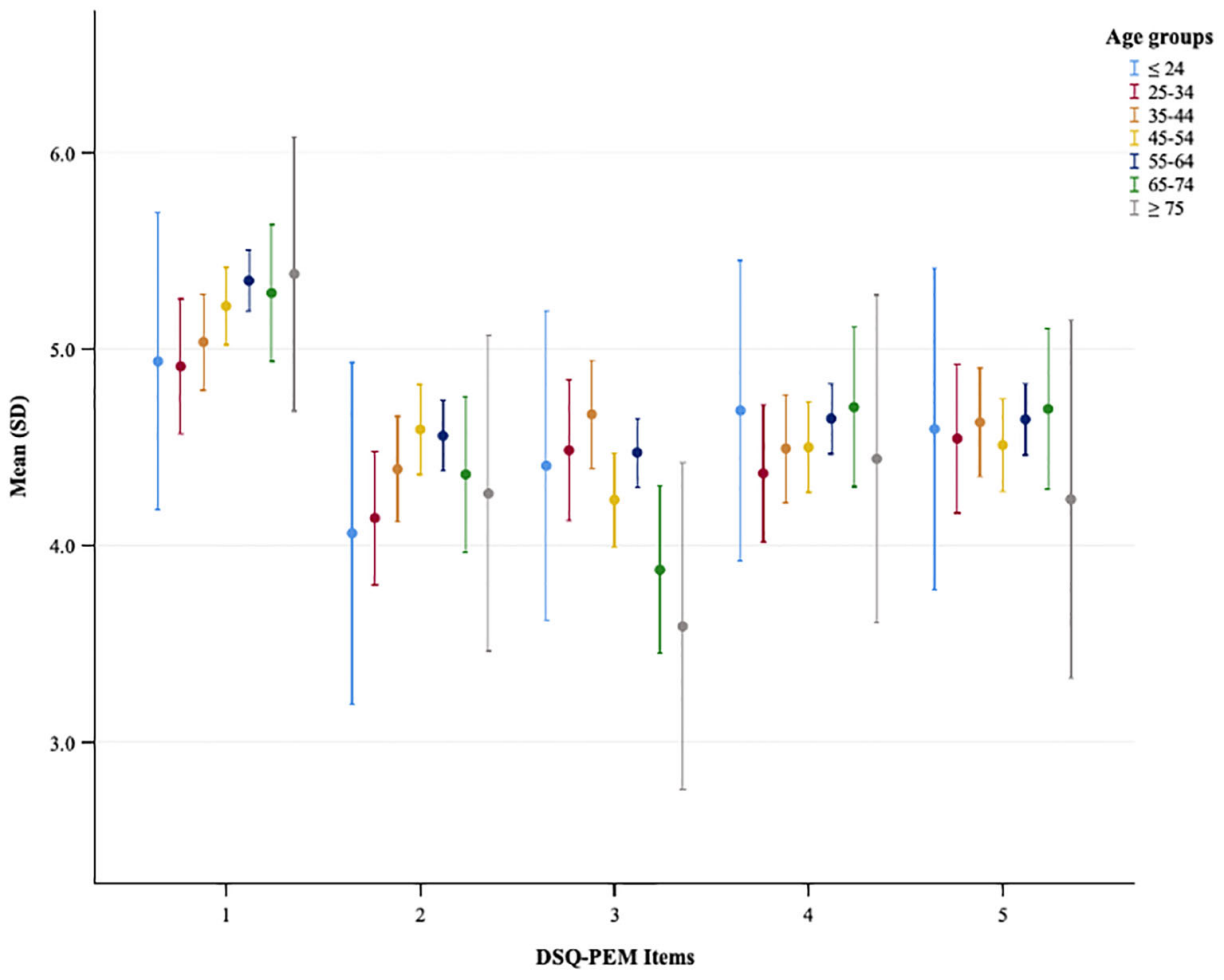
A plot showing the mean and standard deviation of DSQ-PEM items across different age groups in the PCC sample. The x-axis represents DSQ-PEM items numbered one to five, and the y-axis shows the mean score ranging from 0 to 3.0 (possible range 0-8). Error bars display the variability in scores.

In the PCC sample, chi-square analyses showed no significant differences across age groups in terms of the number of positive screenings in scoring step 1; however, a significant difference emerged in scoring step 2 (**Supplementary Table 11**). Kruskal-Wallis tests showed no significant differences among age groups regarding most continuous PEM scores and the extended PEM total score (**Supplementary Tables 12, 13**, **Figure 4**).

## Discussion

The aim of this study was to validate a version of the DSQ-PEM translated into German through a complex pre-translation and back-translation procedure and involving a large, representative German sample and a clinical sample of patients diagnosed with physician-diagnosed PCC.

The results indicate that the first five items of the German version of the DSQ-PEM exhibited high internal consistency, as measured by Cronbach’s alpha, in both the general population sample and the PCC sample. This suggests good reliability of the instrument, thus providing a dependable measurement of PEM.

Regarding the convergent validity of the German version of the DSQ-PEM, a significant correlation was found between the extended PEM total score and the PHQ-4 sum score in both the general population sample and the PCC sample.

This finding is consistent with the results of May et al. (2024) (15) and Sluka et al. (2024) (16), who demonstrated a significant correlation between higher PEM levels and higher depression scores, recorded via the Center of Epidemiologic Studies Depression Scale (CES-D) and the Hospital Anxiety and Depression Scale (HADS-D). In this context, it must be taken into account for the purposes of comparability that the present study employed the PHQ-4 to assess a comprehensive construct of depression and anxiety.

The significant positive correlations between the extended PEM total score and the PHQ-4 measurement instruments in both samples suggest that the constructs of PEM and depression/anxiety may overlap in some aspects. However, the positive correlations were moderate, indicating that they may be relatively distinct, divergent constructs. The latter is supported by the findings from the study conducted by Hawk et al. (2006) (27), which utilized various frequency and severity measures of PEM and other symptoms to differentiate between individuals with CFS and major depression with 100% accuracy. Consequently, PEM appears to be appropriate for distinguishing CFS from other psychiatric or somatic disorders, including major depression. However, further research is recommended to explore the diagnostic differentiation and potential overlaps between CFS, depression and other psychological constructs.

The significant correlation between the extended PEM total score and the Chalder Fatigue Scale score also indicates good convergent validity of the German version of the DSQ-PEM. Again, only a moderately strong positive correlation was observed, which emphasizes the importance of considering PEM as a separate construct from general fatigue/exhaustion.

The known-group validity analyses further demonstrated that the German version of the DSQ-PEM reliably differentiates between individuals from the general population and those with PCC using both the binary scoring method (scoring steps 1 and 2) and the continuous scoring methods (continuous PEM scores, extended PEM total score), thereby distinguishing between samples that are already known to differ in terms of PEM levels.

The regression analyses conducted with more finely graded metrics revealed that, for both samples, gender and educational status were significantly associated with PEM severity, with female gender and lower educational status correlating with higher PEM severity. This aligns with the findings of Jason et al. (1999) (28), who demonstrated, using a US sample of 28,673 adults, the highest rates of CFS in women, minority groups, and those with low educational and occupational status. These results further corroborate the findings of a recent review and meta-analysis conducted by Yoon et al. (2023) (29), which reported a higher prevalence of fatigue in women, as well as the research presented by Engberg et al. (2017) (30), which showed that in the general populace, irrespective of gender, a higher socioeconomic status correlates with lower fatigue scores.

With regard to the association between the extent of PEM and age, different results were found in the general population sample and the PCC. In the general population sample, there was a clear age association; namely, older age was linked to higher PEM levels. This finding is consistent with the study by Schwartz et al. (2003) (31), which showed an almost linear relationship between age and fatigue severity in a representative German sample, with older age being associated with higher fatigue scores.

In the PCC sample, such an age association was not found; however, this aligns with the results of the study by Jason et al. (1999) (28), which showed that CFS occurs primarily in middle-aged groups and tends to be independent of the natural aging process.

In agreement with the study by Jason et al. (1999) (28), the highest number of positive Step 1 screenings in the PCC sample in the present study was found in the age group 55 – 64 years (38.6%), alongside the highest number of positive Step 2 screenings in the age groups 45 – 54 years and 55 – 64 years. The reasons why this age group is particularly affected remain unclear. Both biological and social factors may be regarded as potential causes.

In the present study, the majority of patients from the PCC sample were included, excluding those who demonstrated very mild symptoms or symptoms considered clinically insignificant according to the Chalder Fatigue Scale. Consequently, it can be inferred that the patients in the PCC sample represent a typical severity profile of the disease.

In a study conducted by Twomey et al. (2022) (32) involving patients suffering from long COVID, 95% of participants indicated experiencing symptoms of PEM as assessed by the DSQ-PEM in the first step. However, when stricter criteria were applied in the second step, only 58.7% of the participants tested positive for the condition. In the present study, 87.7% of participants exhibited symptoms of PEM in the first step; however, only 20.8% met the expanded criteria in the second step, which included a stricter duration criterion for PEM. Moreover, a recent study evaluating long COVID patients using the DSQ Short Form and additional DSQ instruments found that 58% of participants fulfilled the case definition for ME/CFS. This finding suggests that our sample in the first step is comparable to that of other studies using standardized instruments to assess PEM in long COVID populations. Conversely, in the second step, the percentage of participants who met the criteria in our PCC sample was lower (20.8%). This observation implies that previously published studies may have included particularly selective samples, predominantly consisting of severely affected individuals. Therefore, the inclusion of an additional validation sample, specifically selecting individuals with severe manifestations of the disease, may be useful to encompass the full spectrum of disease severity. In the context of the present study, the low scoring step 2 prevalence may have resulted in a decrease or underestimation of the screening sensitivity.

A limitation of the study is that no test-retest reliability was calculated, meaning that no conclusions can be drawn about temporal stability or test-retest reliability.

The strengths of the study include the use of a large, population-based, representative sample and the examination of a clinical sample with PCC. By applying and considering different evaluation methods (binary, continuous), the psychometric validity of the German version of the DSQ-PEM could be thoroughly examined.

Nonetheless, it is important to consider that cultural factors may affect how individuals report symptoms and interpret their severity or frequency. Norms related to mental health within different cultures, such as stigmatization and the inclination to underreport, could result in symptoms being described as less pronounced or more cautiously (33–35).

Moreover, there are additional moderating factors, such as affective temperaments (36), that should be considered when examining symptom perception.

In summary, this study confirms the strong psychometric properties of the German version of the DSQ-PEM and endorses the instrument as a reliable and valid tool for measuring PEM in Germany and other German-speaking countries.

## Data Availability

The raw data supporting the conclusions of this article will be made available by the authors, without undue reservation.
