# Supplementary material for: Reliability and validity of the German version of the DePaul Symptom Questionnaire Post-Exertional Malaise (DSQ-PEM)
Source: Front Psychiatry. 2025 Sep 4;16:1647040. doi: 10.3389/fpsyt.2025.1647040 (PMC12443770; doi:10.3389/fpsyt.2025.1647040)
Supplement: Supplementary file 1 [file SupplementaryFile1.docx]

**Supplement**

**German version of the DePaul Symptom Questionnaire Post-Exertional Malaise (DSQ-PEM)**

Markieren Sie bitte jedes der nachstehend aufgeführten Symptome. Füllen Sie bitte die Tabelle von links nach rechts aus. Bitte machen Sie in jeder Zeile zwei Kreuze.

|  | Häufigkeit | | | | | Schweregrad | | | | |
| --- | --- | --- | --- | --- | --- | --- | --- | --- | --- | --- |
|  | **Wie häufig** hatten Sie dieses Symptom in den vergangenen sechs Monaten? | | | | | **Wie stark** hat Sie dieses Symptom in den vergangenen sechs Monaten beeinträchtigt? | | | | |
|  | 0 =  nie | 1 = manchmal | 2 = etwa die Hälfte der Zeit | 3 = meistens | 4 = immer | 0 =  Symptom nicht vorhanden | 1 = leicht | 2= mittel | 3 = stark | 4 = sehr stark |
| Schweres, erschlagendes Gefühl nach Beginn körperlicher Betätigung |  |  |  |  |  |  |  |  |  |  |
| Schmerzen oder Erschöpfung am Tag nach nicht anstrengenden alltäglichen Aktivitäten |  |  |  |  |  |  |  |  |  |  |
| Geistig ermüdet nach geringster Anstrengung |  |  |  |  |  |  |  |  |  |  |
| Körperliche Erschöpfung nach minimaler Bewegung |  |  |  |  |  |  |  |  |  |  |
| Körperlich ausgelaugt oder krank nach leichter Aktivität |  |  |  |  |  |  |  |  |  |  |

| Wenn Sie nach der aktiven Teilnahme an Freizeitaktivitäten, Sport oder Ausflügen mit Freunden erschöpft sind, würden Sie sich innerhalb von 1-2 Stunden nach Ende der Aktivität erholen | ja……………………………………………………………..  nein…………………………………………………………. |
| --- | --- |

Wählen Sie bitte für jede der nachstehenden Fragen diejenige Antwort aus, die Ihren Zustand am besten beschreibt.

| Nehmen Sie eine Zunahme Ihrer Erschöpfung /Ihres Energiemangels nach geringster körperlicher Anstrengung wahr? | ja…………………………………………………………  nein…………………………………………………… |
| --- | --- |

| Nehmen Sie eine Zunahme Ihrer Erschöpfung /Ihres Energiemangels nach geringster geistiger Anstrengung wahr? | ja………………………………………………………….  nein…………………………………………………… |
| --- | --- |

| Wenn Sie sich nach Aktivitäten schlechter fühlen, wie lange dauert das an? | < 1 Stunde……………………………………………  2-3 Stunden………………………………………  4-10 Stunden………………………………………  11-13 Stunden……………………………………  14-23 Stunden ……………………………………  >24 Stunden………………………………………. |
| --- | --- |

| Vermeiden Sie körperliche Betätigung, weil diese Ihre Symptome verschlechtert? | ja………………………………………………………….  nein……………………………………………………… |
| --- | --- |

| Wurde bei Ihnen ein Post-COVID-Syndrom diagnostiziert? | ja……………………………………………………………  nein……………………………………………………… |
| --- | --- |

Kuczyk et al. (2025) Reliability and validity of the German version of the DePaul Symptom Questionnaire Post-Exertional Malaise doi :10.3389/fpsyt.2025.1647040

Original source : Cotler et al. (2018) A brief questionnaire to assess post-exertional malaise doi : 10.3390/diagnostics8030066.
