# Supplementary material for: Reliability and validity of the German version of the DePaul Symptom Questionnaire Post-Exertional Malaise (DSQ-PEM)
Source: Front Psychiatry. 2025 Sep 4;16:1647040. doi: 10.3389/fpsyt.2025.1647040 (PMC12443770; doi:10.3389/fpsyt.2025.1647040)
Supplement: Supplementary file 2 [file SupplementaryFile2.zip › Supplementary Table 1.DOCX]

**Supplementary Table 1**. Results of the regression analyses including the continuous PEM scores as dependent variable and the sample affiliation along with socio-demographic variables as independent variables.

|  | 1. A minimum of exercise makes you physically tired | | | | | 2. Physically drained or sick after mild activity | | | | | | 3. Next-day soreness or fatigue after non-strenuous, everyday activities | | | | | 4. Mentally tired after the slightest exertion | | | | | 5. Dead, heavy feeling after starting to exercise | | | | |
| --- | --- | --- | --- | --- | --- | --- | --- | --- | --- | --- | --- | --- | --- | --- | --- | --- | --- | --- | --- | --- | --- | --- | --- | --- | --- | --- |
|  | **B** | **SE** | **β** | **t** | **p** | **B** | **SE** | **β** | **T** | **p** | **B** | | **SE** | **β** | **t** | **p** | **B** | **SE** | **β** | **t** | **p** | **B** | **SE** | **β** | **t** | **p** |
| **Sample** | 4.07 | .06 | .74 | 66.30 | <.001 | 3.35 | .06 | .65 | 51.54 | < .001 | 3.59 | | .06 | .70 | 57.63 | < .001 | 3.68 | .06 | .70 | 58.00 | < .001 | 3.78 | .06 | .71 | 59.18 | < .001 |
| **Sex** | 0.13 | .06 | .02 | 2.22 | .027 | 0.32 | .06 | .06 | 5.10 | < .001 | 0.26 | | .06 | .05 | 4.18 | < .001 | 0.22 | .06 | .04 | 3.51 | < .001 | 0.22 | .06 | .04 | 3.43 | < .001 |
| **Age in Years** | 0.02 | .00 | .15 | 13.30 | <.001 | .0.02 | .00 | .14 | 11.55 | < .001 | 0.01 | | .00 | .06 | 4.74 | <.001 | 0.02 | .00 | .11 | 9.60 | < .001 | 0.02 | .00 | .10 | 8.24 | < .001 |
| **Education** | -0.17 | .07 | -.03 | -2.42 | .016 | -0.06 | .08 | -.01 | -.78 | .436 | -0.03 | | .07 | .00 | -.38 | .706 | -0.16 | .07 | -.03 | -2.20 | .028 | -0.15 | .07 | -.02 | -2.04 | .041 |
