# Supplementary material for: Reliability and validity of the German version of the DePaul Symptom Questionnaire Post-Exertional Malaise (DSQ-PEM)
Source: Front Psychiatry. 2025 Sep 4;16:1647040. doi: 10.3389/fpsyt.2025.1647040 (PMC12443770; doi:10.3389/fpsyt.2025.1647040)
Supplement: Supplementary file 2 [file SupplementaryFile2.zip › Supplementary Table 2.docx]

**Supplementary Table 2**. Gender comparison in the general population sample with regard to binary PEM scores (Scoring Steps 1 and 2). The figures n (%) indicate the number of positive screenings.

|  | General population sample  (**n = 2263)** | | Chi-Square-test  (χ², df, p-value) |
| --- | --- | --- | --- |
|  | Female  N=1162 | Male  N=1100 |  |
| 1. A minimum of exercise makes you physically tired, n(%) | 103 (8.9) | 93 (8.5) | χ² =.89  df = 2  p = .892 |
| 2. Physically drained or sick after mild activity, n(%) | 108 (9.4) | 78 (7.1) | χ² = 3.77  df = 2  p = .152 |
| 3. Next-day soreness or fatigue after non-strenuous, everyday activities, n(%) | 70 (6.1) | 58 (5.3) | χ²= 0.71  df = 2  p = .700 |
| 4. Mentally tired after the slightest exertion, n(%) | 82 (7.1) | 69 (6.3) | χ² = 0.66  df = 2  p = .718 |
| 5. Dead, heavy feeling after starting to exercise, n(%) | 74 (6.4) | 68 (6.2) | χ² = 0.10  df = 2  p = .949 |
| Scoring step 1, n(%) | 165 (14.3) | 121 (11.1) | χ² = 5.521  df = 2  p = .063 |
| 7 & 8. Do you experience a worsening of your fatigue/energy-related illness after engaging in minimal physical and/or mental effort? n(%) | 389 (33.6) | 286 (26.1) | χ² = 15.41  df = 2  p < .001 |
| 9. Duration 14-23 hours or > 24 hours, n(%) | 12 (1.1) | 11(1.1) | χ² = 0.02  df = 2  p = .992 |
| Scoring Step 2, n(%) | 10 (0.9) | 8 (0.7) | χ² = 0.13  df = 2  p = .935 |
