# Supplementary material for: Reliability and validity of the German version of the DePaul Symptom Questionnaire Post-Exertional Malaise (DSQ-PEM)
Source: Front Psychiatry. 2025 Sep 4;16:1647040. doi: 10.3389/fpsyt.2025.1647040 (PMC12443770; doi:10.3389/fpsyt.2025.1647040)
Supplement: Supplementary file 2 [file SupplementaryFile2.zip › Supplementary Table 3.DOCX]

**Supplementary Table 3.** Gender comparison in the general population sample with regard to the continuous PEM scores.

|  |  | General population sample  (**N = 2263)** | | Mann-Whitney U test (Z, p- value) |
| --- | --- | --- | --- | --- |
|  |  | Female  **N=1162** | Male  **N=1100** |  |
| 1. A minimum of exercise makes you physically tired | M (SD) | 1.15 (1.66) | 1.01 (1.66) | Z = 2.62  p = .009 |
|  | Median (IQR) | 0.00 (2.00) | 0.0 (2.00) |  |
| 2. Physically drained or sick after mild activity | M (SD) | 1.18 (1.74) | 0.92 (1.58) | Z= -3.96  p < .001 |
|  | Median (IQR) | 0.0 (2.00) | 0.0 (2.00) |  |
| 3. Next-day soreness or fatigue after non-strenuous, everyday activities | M (SD) | 0.84 (1.47) | 0.65 (1.35) | Z= 3.52  p <.001 |
|  | Median (IQR) | 0.0 (2.00) | 0.0 (0.00) |  |
| 4. Mentally tired after the slightest exertion | M (SD) | 0.89 (1.56) | 0.76 (1.51) | Z= -2.77  p = .006 |
|  | Median (IQR) | 0.0 (2.00) | 0.0 (1.00) |  |
| 5. Dead, heavy feeling after starting to exercise | M (SD) | 0.85 (1.52) | 0.72 (1.49) | Z= 2.55  p = .011 |
|  | Median (IQR) | 0.0 (2.00) | 0.0 (0.0) |  |
