# Supplementary material for: Reliability and validity of the German version of the DePaul Symptom Questionnaire Post-Exertional Malaise (DSQ-PEM)
Source: Front Psychiatry. 2025 Sep 4;16:1647040. doi: 10.3389/fpsyt.2025.1647040 (PMC12443770; doi:10.3389/fpsyt.2025.1647040)
Supplement: Supplementary file 2 [file SupplementaryFile2.zip › Supplementary Table 4.DOCX]

**Supplementary Table 4.** Gender comparison in the general population sample with regard to the extended PEM total score (0-46).

|  | General population sample  (**n = 2263)** | | Mann-Whitney U test (Z, p- value) |
| --- | --- | --- | --- |
|  | Female  **N=1162** | Male  **N=1100** |  |
| Mean (SD) | 6.70 (7.65) | 5.77 (7.57) | Z = -4.021  p < .001 |
| Median (IQR) | 3.0 (9.0) | 2.0 (7.0) |  |
