# Supplementary material for: Reliability and validity of the German version of the DePaul Symptom Questionnaire Post-Exertional Malaise (DSQ-PEM)
Source: Front Psychiatry. 2025 Sep 4;16:1647040. doi: 10.3389/fpsyt.2025.1647040 (PMC12443770; doi:10.3389/fpsyt.2025.1647040)
Supplement: Supplementary file 2 [file SupplementaryFile2.zip › Supplementary Table 5.docx]

**Supplementary Table 5.** Comparison of gender in the PCC sample with regard to the binary PEM scores (including Scoring Steps 1 and 2). The figures n (%) indicate the number of positive screenings.

|  | PCC sample  **(N= 1448)** | | Chi-Square-test  (χ², df, p-value) |
| --- | --- | --- | --- |
|  | Female  N=1038 | Male  N=410 |  |
| 1. A minimum of exercise makes you physically tired, n (%) | 777 (76.0) | 314 (76.8) | χ² = 0.09  df = 1  p = .765 |
| 2. Physically drained or sick after mild activity, n (%) | 624 (61.1) | 244 (59.7) | χ² = .261  df = 1  p = .610 |
| 3. Next-day soreness or fatigue after non-strenuous, everyday activities, n (%) | 640 (62.6) | 236 (57.7) | χ²= 2.979  df = 1  p = .084 |
| 4. Mentally tired after the slightest exertion, n (%) | 657 (64.3) | 258 (63.1) | χ² = 0.184  df = 1  p = .668 |
| 5. Dead, heavy feeling after starting to exercise, n (%) | 666 (65.2) | 252 (61.6) | χ² = 1.603  df = 1  p = .205 |
| Scoring Step 1, n (%) | 900 (88.1) | 354 (86.6) | χ² = 0.689  df = 1  p = .406 |
| 7 & 8. Do you experience a worsening of your fatigue/ energy-related illness after engaging in minimal physical and/or mental effort? n (%) | 884 (86.4) | 343 (83.9) | χ² = 1.548  df = 1  p = .213 |
| 9. Duration 14-23 hours or > 24 hours, n (%) | 271 (26.5) | 58 (14.2) | χ² = 25.19  df = 1  p < .001 |
| Scoring Step 2, n (%) | 246 (24.1) | 52 (12.7) | χ² = 22.93  df = 1  p < .001 |
