# Supplementary material for: Reliability and validity of the German version of the DePaul Symptom Questionnaire Post-Exertional Malaise (DSQ-PEM)
Source: Front Psychiatry. 2025 Sep 4;16:1647040. doi: 10.3389/fpsyt.2025.1647040 (PMC12443770; doi:10.3389/fpsyt.2025.1647040)
Supplement: Supplementary file 2 [file SupplementaryFile2.zip › Supplementary Table 6.DOCX]

**Supplementary Table 6.** Gender comparison in the PCC sample with regard to continuous PEM scores.

|  |  | PCC sample  **(N= 1448)** | | Mann-Whitney U test (Z, p- value) |
| --- | --- | --- | --- | --- |
|  |  | Female  N=1038 | Male  N=410 |  |
| 1. A minimum of exercise makes you physically tired | M (SD) | 5.17 (1.89) | 5.30 (1.85) | Z = -1.39  p = .163 |
|  | Median (IQR) | 5.00 (2.00) | 6.00 (3.00) |  |
| 2. Physically drained or sick after mild activity | M (SD) | 4.54 (2.07) | 4.34 (2.06) | Z = 1.41  p = .159 |
|  | Median (IQR) | 5.00 (3.00) | 4.00 (3.00) |  |
| 3. Next-day soreness or fatigue after non-strenuous, everyday activities | M (SD) | 4.46 (2.12) | 4.21 (2.18) | Z= 1.821  p = .069 |
|  | Median (IQR) | 4.00 (3.00) | 4.00 (3.50) |  |
| 4. Mentally tired after the slightest exertion | M (SD) | 4.63 (2.11) | 4.42 (3.00) | Z= 1.522  p = .128 |
|  | Median (IQR) | 5.00 (3.00) | 50.0 (37.50) |  |
| 5. Dead, heavy feeling after starting to exercise | M (SD) | 4.67 (2.18) | 4.42 (2.12) | Z= 1.994  p = .046 |
|  | Median (IQR) | 5.00 (3.00) | 4.00 (3.00) |  |
