# Supplementary material for: Reliability and validity of the German version of the DePaul Symptom Questionnaire Post-Exertional Malaise (DSQ-PEM)
Source: Front Psychiatry. 2025 Sep 4;16:1647040. doi: 10.3389/fpsyt.2025.1647040 (PMC12443770; doi:10.3389/fpsyt.2025.1647040)
Supplement: Supplementary file 2 [file SupplementaryFile2.zip › Supplementary Table 7.DOCX]

**Supplementary Table 7.** Comparison of gender in the PCC sample with regard to the extended PEM total score.

|  | PCC sample  **(N= 1448)** | | Mann-Whitney U test (Z, p-value) |
| --- | --- | --- | --- |
|  | Female  **N=1038** | Male  **N=410** |  |
| Mean (SD) | 26.80 (9.70) | 25.47 (9.38) | Z = -2.06  p = .040 |
