# Supplementary material for: Reliability and validity of the German version of the DePaul Symptom Questionnaire Post-Exertional Malaise (DSQ-PEM)
Source: Front Psychiatry. 2025 Sep 4;16:1647040. doi: 10.3389/fpsyt.2025.1647040 (PMC12443770; doi:10.3389/fpsyt.2025.1647040)
Supplement: Supplementary file 2 [file SupplementaryFile2.zip › Supplementary Table 8.docx]

|  | General population sample  (**N = 2263)** | | | | | | |  |
| --- | --- | --- | --- | --- | --- | --- | --- | --- |
|  | **≤ 24** | **25-34** | **35-44** | **45-54** | **55-64** | **65-74** | **≥ 75** | Chi-Square-test  (χ², df, p-value) |
| 1. A minimum of exercise makes you physically tired, n (%) | 5 (2.6) | 9 (4.6) | 13 (6.6) | 24 (12.2) | 38 (19.4) | 55 (28.1) | 52 (26.5) | χ² = 156.51  (df = 6)  p < .001 |
| 2. Physically drained or sick after mild activity, n (%) | 9 (4.8) | 11 (5.9) | 12 (6.5) | 20 (10.8) | 43 (23.1) | 52 (28.0) | 39 (21.0) | χ² = 95.68  (df = 6)  p < .001 |
| 3. Next-day soreness or fatigue after non-strenuous, everyday activities, n (%) | 11 (8.6) | 10 (7.8) | 13 (10.2) | 16 (12.5) | 30 (23.4) | 25 (19.5) | 23 (18.0) | χ² = 30.38  df = 6  p < .001 |
| 4. Mentally tired after the slightest exertion, n (%) | 8 (5.3) | 9 (6.0) | 13 (8.6) | 13 (8.6) | 34 (22.5) | 40 (26.5) | 34 (22.5) | χ² = 79.80,  df = 6  p < .001 |
| 5. Dead, heavy feeling after starting to exercise, n (%) | 6 (4.2) | 8 (5.6) | 12 (8.5) | 12 (8.5) | 35 (24.6) | 37 (26.1) | 32 (22.5) | χ² =77.85  df=6  p < .001 |
| Scoring Step 1, n (%) | 17 (5.9) | 18 (6.3) | 23 (8.0) | 34 (11.9) | 59 (20.6) | 74 (25.9) | 61 (21.3) | χ² = 135.32  df = 6  p < .001 |
| 7 & 8. Do you experience a worsening of your fatigue/ energy-related illness after engaging in minimal physical and/or mental effort? n (%) | 29 (4.3) | 59 (8.7) | 74 (11.0) | 99 (14.7) | 149 (22.1) | 163 (24.1) | 102 (15.1) | χ² =175.25  df = 6  p < .001 |
| 9. Duration 14-23 hours or > 24 hours, n (%) | 4 (17.4) | 0 (0.0) | 3 (13.0) | 2 (8.7) | 10 (43.5) | 0 (0.0) | 4 (17.4) | χ² = 19.96  df = 6  p = .003 |
| Scoring Step 2, n (%) | 1 (5.6) | 0 (0.0) | 3 (16.7) | 1 (5.6) | 9 (50.0) | 0 (0.0) | 4 (22.2) | χ² = 20.85  df = 6  p = .002 |

**Supplementary Table 8.** Age group comparisons in the general population sample with regard to binary PEM scores. The figures n (%) indicate the number of positive screenings.
