# Supplementary material for: Reliability and validity of the German version of the DePaul Symptom Questionnaire Post-Exertional Malaise (DSQ-PEM)
Source: Front Psychiatry. 2025 Sep 4;16:1647040. doi: 10.3389/fpsyt.2025.1647040 (PMC12443770; doi:10.3389/fpsyt.2025.1647040)
Supplement: Supplementary file 2 [file SupplementaryFile2.zip › Supplementary Table 9.DOCX]

**Supplementary Table 9.** Comparisons of age groups in the general population sample with regard to continuous PEM scores.

|  |  | General population sample  (N **= 2263)** | | | | | | |  |
| --- | --- | --- | --- | --- | --- | --- | --- | --- | --- |
|  |  | **≤ 24** | **25-34** | **35-44** | **45-54** | **55-64** | **65-74** | **≥ 75** | Kruskal-Wallis test |
| 1. A minimum of exercise makes you physically tired | M (SD) | 0.55 (1.24) | 0.60 (1.19) | 0.62 (1.24) | 0.97 (1.54) | 1.24 (1.78) | 1.61 (1.85) | 2.32 (2.05) | H(6) = 215.97,  p < .001 |
|  | Mdn (IQR) | 0.0 (0.0) | 0.0 (0.0) | 0.0 (0.0) | 0.0 (2.0) | 0.0 (2.0) | 2.0 (2.0) | 2.0 (4.0) |  |
| 2. Physically drained or sick after mild activity | M (SD) | 0.69 (1.48) | 0.55 (1.16) | 0.59 (1.23) | 0.88 (1.55) | 1.25 (1.83) | 1.58 (1.88) | 2.17 (1.92) | H(6) = 209.93,  p < .001 |
|  | Mdn (IQR) | 0.0 (0.0) | 0.0 (0.0) | 0.0 (0.0) | 0.0 (2.0) | 0.0 (2.00) | 1.00 (2.00) | 2.0 (3.0) |  |
| 3. Next-day soreness or fatigue after non-strenuous, everyday activities | M (SD) | 0.65 (1.56) | 0.43 (1.06) | 0.48 (1.18) | 0.69 (1.40) | 0.84 (1.54) | 0.97 (1.43) | 1.45 (2.75) | H(6) = 110.94,  p < .001 |
|  | Mdn (IQR) | 0.0 (0.0) | 0.0 (0.0) | 0.0 (0.0) | 0.0 (0.75) | 0.0 (2.00) | 0.0 (2.00) | 1.00 (2.75) |  |
| 4. Mentally tired after the slightest exertion | M (SD) | 0.47 (1.26) | 0.41 (1.06) | 0.43 (1.12) | 0.69 (1.41) | 0.96 (1.72) | 1.27 (1.72) | 1.85 (1.90) | H(6) = 205.79,  p < .001 |
|  | Mdn (IQR) | 0.0 (0.0) | 0.0 (0.0) | 0.0 (0.0) | 0.0 (1.0) | 0.0 (2.0) | 0.0 (2.0) | 2.0 (3.0) |  |
| 5. Dead, heavy feeling after starting to exercise | M (SD) | 0.44 (1.26) | 0.39 (1.01) | 0.49 (1.23) | 0.66 (1.39) | 0.89 (1.68) | 1.14 (1.70) | 1.78 (1.82) | H(6) = 173.20,  p < .001 |
|  | Mdn (IQR) | 0.0 (0.0) | 0.0 (0.0) | 0.0 (0.0) | 0.0 (0.0) | 0.0 (2.0) | 0.0 (2.0) | 2.0 (3.0) |  |
