# Supplementary material for: Reliability and validity of the German version of the DePaul Symptom Questionnaire Post-Exertional Malaise (DSQ-PEM)
Source: Front Psychiatry. 2025 Sep 4;16:1647040. doi: 10.3389/fpsyt.2025.1647040 (PMC12443770; doi:10.3389/fpsyt.2025.1647040)
Supplement: Supplementary file 2 [file SupplementaryFile2.zip › Supplementary Table 10.DOCX]

**Supplementary Table 10.** Age group comparisons in the general population sample with regard to the extended PEM total score.

|  | General population sample  (**N = 2263)** | | | | | | |  |
| --- | --- | --- | --- | --- | --- | --- | --- | --- |
|  | **≤ 24** | **25-34** | **35-44** | **45-54** | **55-64** | **65-74** | **≥ 75** | Kruskal-Wallis test |
| M(SD) | 4.14 (6.66) | 3.76 (5.18) | 4.11 (5.95) | 5.56 (7.0) | 7.10 (8.55) | 8.65 (7.94) | 12.33 (8.87) | H(6) = 280.82,  p < .001 |
| Median (IQR) | 1.0 (2.75) | 1.0 (3.0) | 1.0 (3.0) | 2.0 (7.0) | 3.0 (9.0) | 7.0 (10.0) | 12.0 (13.0) |  |
