# Supplementary material for: Reliability and validity of the German version of the DePaul Symptom Questionnaire Post-Exertional Malaise (DSQ-PEM)
Source: Front Psychiatry. 2025 Sep 4;16:1647040. doi: 10.3389/fpsyt.2025.1647040 (PMC12443770; doi:10.3389/fpsyt.2025.1647040)
Supplement: Supplementary file 2 [file SupplementaryFile2.zip › Supplementary Table 11.docx]

|  | PCC sample **(N = 1448)** | | | | | | |  |
| --- | --- | --- | --- | --- | --- | --- | --- | --- |
|  | **≤ 24** | **25-34** | **35-44** | **45-54** | **55-64** | **65-74** | **≥ 75** | Chi-Square-test  (χ²) |
| 1. A minimum of exercise makes you physically tired, n (%) | 21 (1.9) | 97 (8.9) | 184 (16.9) | 265 (24.3) | 416 (38.1) | 82 (7.5) | 26 (2.4) | χ² = 6.88  df = 6  p = .332 |
| 2. Physically drained or sick after mild activity, n (%) | 17 (2.0) | 72 (8.3) | 137 (15.8) | 228 (26.3) | 341 (39.3) | 56 (6.5) | 17 (2.0) | χ² = 17.71  df = 6  p = .007 |
| 3. Next-day soreness or fatigue after non-strenuous, everyday activities, n (%) | 19 (2.2) | 80 (9.1) | 164 (18.7) | 213 (24.3) | 335 (38.2) | 51 (5.8) | 14 (1.6) | χ² = 17.38  df = 6  p = .008 |
| 4. Mentally tired after the slightest exertion, n (%) | 21 (2.3) | 81 (8.9) | 155 (16.9) | 221 (24.2) | 346 (37.8) | 70 (7.7) | 21 (2.3) | χ² = 2.65  df = 6  p = .852 |
| 5. Dead, heavy feeling after starting to exercise, n (%) | 21 (2.3) | 86 (9.4) | 158 (17.2) | 221 (24.1) | 349 (38.0) | 65 (7.1) | 18 (2.0) | χ² = 3.49  df = 6  p = .745 |
| Scoring Step 1, n (%) | 29 (2.3) | 120 (9.6) | 218 (17.4) | 308 (24.6) | 457 (36.4) | 94 (7.5) | 28 (2.2) | χ² = 1.79  df = 6  p = .938 |
| 7 & 8. Do you experience a worsening of your fatigue/energy-related illness after engaging in minimal physical and/or mental effort? n (%) | 28 (2.3) | 119 (9.7) | 213 (17.4) | 299 (24.4) | 453 (36.9) | 90 (7.3) | 25 (2.0) | χ² = 4.85  df = 6  p = .564 |
| 9. Duration 14-23 hours or > 24 hours, n (%) | 1 (0.3) | 31 (9.4) | 79 (24.0) | 100 (30.4) | 104 (31.6) | 9 (2.7) | 5 (1.5) | χ² = 41.04  df = 6  p < .001 |
| Scoring Step 2, n (%) | 1 (0.3) | 26 (8.7) | 73 (24.5) | 95 (31.9) | 90 (30.2) | 9 (3.0) | 4 (1.3) | χ² = 41.58  df = 6  p < .001 |

**Supplementary Table 11.** Age group comparisons in the PCC sample with regard to binary PEM scores. The figures n (%) indicate the number of positive screenings.
