# Supplementary material for: Reliability and validity of the German version of the DePaul Symptom Questionnaire Post-Exertional Malaise (DSQ-PEM)
Source: Front Psychiatry. 2025 Sep 4;16:1647040. doi: 10.3389/fpsyt.2025.1647040 (PMC12443770; doi:10.3389/fpsyt.2025.1647040)
Supplement: Supplementary file 2 [file SupplementaryFile2.zip › Supplementary Table 12.DOCX]

**Supplementary Table 12.** Age group comparisons in the PCC sample with regard to continuous PEM scores.

|  |  | PCC sample **(N = 1448)** | | | | | | |  |
| --- | --- | --- | --- | --- | --- | --- | --- | --- | --- |
|  |  | **≤ 24** | **25-34** | **35-44** | **45-54** | **55-64** | **65-74** | **≥ 75** | Kruskal-Wallis test |
| 1. A minimum of exercise makes you physically tired | M (SD) | 4.94 (2.09) | 4.91 (2.03) | 5.04 (1.95) | 5.22 (1.87) | 5.35 (1.80) | 5.29 (1.80) | 5.38 (2.00) | H(6) = 8.50,  p = .204 |
|  | Median (IQR) | 5.0 (3.0) | 5.0 (3.0) | 5.0 (2.0) | 5.0 (2.0) | 6.0 (3.0) | 5.0 (3.0) | 6.0 (3.0) |  |
| 2. Physically drained or sick after mild activity | M (SD) | 4.06 (2.41) | 4.14 (2.00) | 4.39 (2.15) | 4.63 (2.06) | 4.58 (2.02) | 4.36 (2.06) | 4.26 (2.30) | H(6) = 9.91,  p = .128 |
|  | Median (IQR) | 4.0 (4.0) | 4.0 (3.0) | 4.0 (3.0) | 5.0 (3.0) | 5.0 (3.0) | 4.0 (3.0) | 4.0 (3.25) |  |
| 3. Next day soreness or fatigue after non-strenuous, everyday activities | M (SD) | 4.41 (2.18) | 4.49 (2.10) | 4.67 (2.19) | 4.27 (2.17) | 4.47 (2.05) | 3.88 (2.20) | 3.59 (2.39) | H(6) = 17.40,  p = .008 |
|  | Median (IQR) | 4.0 (3.75) | 4.0 (3.0) | 5.0 (3.0) | 4.0 (3.0) | 5.0 (3.0) | 3.0 (4.0) | 3.0 (4.0) |  |
| 4. Mentally tired after the slightest exertion | M (SD) | 4.69 (2.12) | 4.37 (2.06) | 4.49 (2.19) | 4.54 (2.09) | 4.65 (2.08) | 4.70 (2.09) | 4.44 (2.39) | H(6) = 2.90,  p = .827 |
|  | Median (IQR) | 5.0 (3.0) | 4.0 (3.0) | 5.0 (3.0) | 5.0 (3.0) | 5.0 (3.0) | 5.0 (3.0) | 4.5 (4.0) |  |
| 5 Dead, heavy feeling after starting to exercise | M (SD) | 4.59 (2.27) | 4.54 (2.23) | 4.63 (2.20) | 4.55 (2.15) | 4.64 (2.12) | 4.70 (2.10) | 4.24 (2.61) | H(6) = 1.10,  p = .983 |
|  | Median (IQR) | 5.0 (3.0) | 5.0 (3.0) | 5.0 (3.0) | 4.0 (3.0) | 5.0 (3.0) | 5.0 (3.0) | 4.0 (4.0) |  |
