# Supplementary material for: Reliability and validity of the German version of the DePaul Symptom Questionnaire Post-Exertional Malaise (DSQ-PEM)
Source: Front Psychiatry. 2025 Sep 4;16:1647040. doi: 10.3389/fpsyt.2025.1647040 (PMC12443770; doi:10.3389/fpsyt.2025.1647040)
Supplement: Supplementary file 2 [file SupplementaryFile2.zip › Supplementary Table 13.DOCX]

**Supplementary Table 13.** Age group comparisons in the PCC sample with regard to the extended PEM total score.

|  | PCC sample **(N = 1448)** | | | | | | |  |
| --- | --- | --- | --- | --- | --- | --- | --- | --- |
|  | **≤ 24** | **25-34** | **35-44** | **45-54** | **55-64** | **65-74** | **≥ 75** | Kruskal-Wallis test |
| Mean (SD) | 25.19 (9.48) | 25.54 (9.27) | 26.72 (10.23) | 26.60 (9.77) | 26.76 (9.30) | 25.52 (9.31) | 24.62 (11.0) | H(6) = 4.77,  p = .573 |
| Median (IQR) | 24 (14.25) | 26 (14.0) | 26 (16.0) | 26 (15.0) | 27 (13.0) | 24 (14.5) | 24 (17.75) |  |
