# Supplementary material for: Reliability and validity of the German version of the DePaul Symptom Questionnaire Post-Exertional Malaise (DSQ-PEM)
Source: Front Psychiatry. 2025 Sep 4;16:1647040. doi: 10.3389/fpsyt.2025.1647040 (PMC12443770; doi:10.3389/fpsyt.2025.1647040)
Supplement: Supplementary file 2 [file SupplementaryFile2.zip › Supplementary Figures 1-2.DOCX]

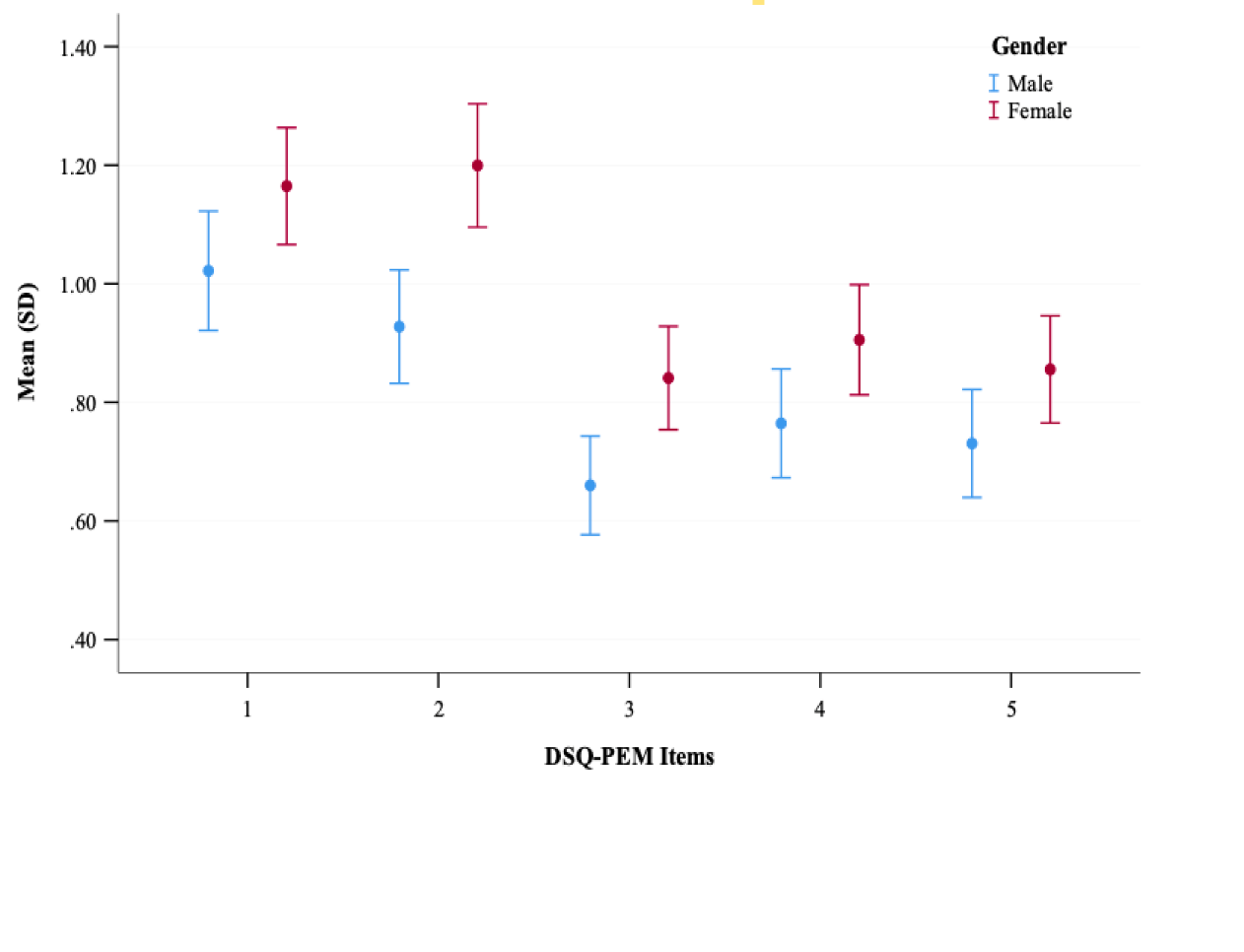


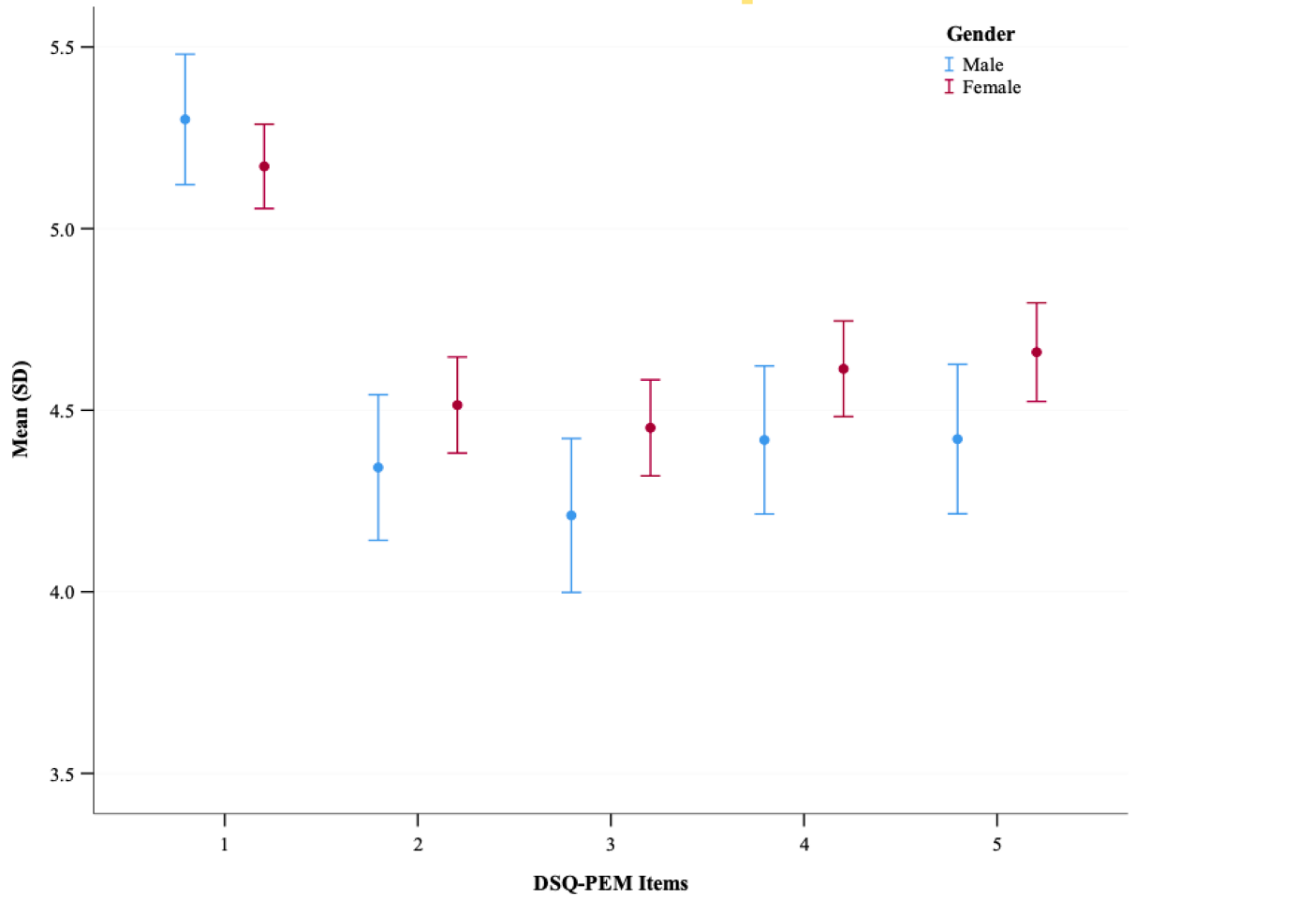


**Supplementary Figure 2.** Means and standard deviations of the sum of frequency and severity (Continuous PEM scores) (ranging from 0 to 8) for the first five items of the DSQ-PEM, stratified by gender in the PCC sample.

**Supplementary Figure 1.** Means and standard deviations of the sum of frequency and severity (Continuous PEM scores) (ranging from 0 to 8) for the first five items of the DSQ-PEM, stratified by gender in the general population sample.
